# Neuroplasticity enables bio-cultural feedback in Paleolithic stone-tool making

**DOI:** 10.1038/s41598-023-29994-y

**Published:** 2023-02-18

**Authors:** Erin Elisabeth Hecht, Justin Pargeter, Nada Khreisheh, Dietrich Stout

**Affiliations:** 1 Department of Human Evolutionary Biology, Harvard University, Cambridge, MA USA; 2 Department of Anthropology, New York University, New York, NY USA; 3 Paleo-Research Institute, University of Johannesburg, Auckland Park, Johannesburg, South Africa; 4 Ancient Technology Center, Cranborne, Dorset UK; 5 Department of Anthropology, Emory University, Atlanta, GA USA

**Keywords:** Anthropology, Archaeology, Cultural evolution, Evolution, Neuroscience, Cognitive neuroscience

## Abstract

Stone-tool making is an ancient human skill thought to have played a key role in the bio-cultural co-evolutionary feedback that produced modern brains, culture, and cognition. To test the proposed evolutionary mechanisms underpinning this hypothesis we studied stone-tool making skill learning in modern participants and examined interactions between individual neurostructural differences, plastic accommodation, and culturally transmitted behavior. We found that prior experience with other culturally transmitted craft skills increased both initial stone tool-making performance and subsequent neuroplastic training effects in a frontoparietal white matter pathway associated with action control. These effects were mediated by the effect of experience on pre-training variation in a frontotemporal pathway supporting action semantic representation. Our results show that the acquisition of one technical skill can produce structural brain changes conducive to the discovery and acquisition of additional skills, providing empirical evidence for bio-cultural feedback loops long hypothesized to link learning and adaptive change.

## Introduction

Stone-tool making has long been recognized as a distinctive human behavior and essential Paleolithic survival skill that likely helped drive hominin brain and behavioral evolution^1–3^. However, the processes of neuroevolutionary change leading to increased tool making abilities in the human lineage has been challenging to study, because while our ancestors’ tools persist in the archaeological record, their brains do not. Longstanding hypotheses linking tool making to the evolution of neural foundations for human culture, cognition, and language^3–6^ have thus been difficult to test.

To address this challenge, researchers have increasingly turned to behavioral experiments with modern participants in order to identify the learning demands^7–12^ (e.g., teaching^9^, perceptual-motor coordination^12^, self-control^10^) and underlying neural mechanisms^13–21^ associated with stone-tool making methods known from the archaeological record. Results generally support the intuition that increasingly complex Paleolithic tool making would have placed increasing demands on hominin perceptual-motor^18,22^, cognitive control^17,19^, and working memory^23^ capacities including complex action sequencing and observational understanding abilities relevant to the evolution of language^16,20,24^. However, it has remained unclear exactly how such increasing functional demands might have translated into observed evolutionary changes in the human brain. We thus sought to ground this research program with respect to known mechanisms of evolutionary change by using Diffusion Tensor Imaging (DTI) to measure variation in brain structure and neuroplastic accommodation associated with differences in initial aptitude and subsequent learning of stone-tool making skills.

Our study focused on the production of “Acheulean” stone tools, as the emergence of this technology has long been regarded as a watershed in human cognitive and cultural evolution^2,25–27^. The most characteristic artifact of this technology, now dated to just over 1.7 million years ago^26,27^, is the teardrop-shaped Achuelean handaxe which is believed to have functioned as a large (> 10 cm), hand-held, cutting tool for butchery and other purposes. In contrast to the earlier technologies, Achuelean handaxe production clearly involves intentional shaping of the stone into a desired form reflecting functional and possibly also esthetic and/or cultural constraints^25,28^. This imposition of intended form requires more complex action sequences^20^ characterized by a nested structure of contingent goals and sub-goals^29^, as well as increased perceptual-motor precision^12^ to reliably achieve desired effects allowing for successful execution of these contingent sequences.

These behavioral observations are supported by functional neuroimaging studies comparing Acheulean tool making to earlier (“Oldowan”^6,25^) techniques. FDG-PET^14^ and functional near infrared spectroscopy (fNIRS)^18,19^ studies of action execution, as well as fMRI studies of action observation^15,20^ and technological judgements on stone tool stimuli^17^ consistently indicate that Acheulean technology causes greater activation in inferior parietal and prefrontal cortex, including especially the right inferior frontal gyrus (rIFG)^14,15,18,20^. This later result is consistent with apparent role of rIFG in complex action control^30,31^ and Stout et al.^20^ found that posterior rIFG response to tool making specifically correlated with the structural complexity of observed action sequences quantified using hidden Markov Modeling and Context Free Grammar extraction methods. These results have been used to argue^14,24,32,33^ that selection for the increasingly complex action organization capacities exemplified by Paleolithic stone technologies would have contributed to the evolution of more general sequence processing capacities, such as chunk-based learning, that are relevant to skill acquisition across a wide range of behavioral domains^34,35^ including language^36^.

The likelihood that the cultural evolution of Paleolithic technologies stimulated and/or was enabled by hominin brain evolution^3,37^ is supported by evidence of evolutionarily derived functionality in the modern human brain regions that are typically recruited by Paleolithic stone-tool making. These include occipital and parietal regions that show novel sensitivity to 3D visual^38^ and tool-use^39,40^ stimuli in humans as compared to macaque monkeys, as well as evolutionarily expanded^41^ prefrontal regions supporting enhanced human action organization and cognitive control. Hecht et al.^42^ used FDG-PET to study object-direction action observation in chimpanzees and humans and found that, whereas both species activated dorsolateral prefrontal cortex, humans showed significantly more activation in regions of inferior parietal, ventral premotor, and inferior temporal cortex also commonly activated by stone-tool making^13–15^. Hecht et al. argued that this reflects additional sensitivity to action details and reliance on bottom-up processing in humans as compared to more coarse-grained goal representation and top-down control strategies in chimpanzees. Such sensitivity to fine perceptual-motor details is crucial to the acquisition and practice of demanding manual skills like stone-tool making^12,43^. In contrast, dorsal prefrontal activity in response to stone-tool making has only been observed in conditions where abstract goal representation is prioritized, such as early stage learning^19^, learning in the absence of instruction^18^, and strategic judgement in the absence of execution^17^.

More broadly, comparative neuroanatomical evidence indicates that frontoparietal systems involved in action observation/execution, including stone-tool making, have been greatly elaborated over the course of human evolution^22^. In macaques, the relevant circuitry is dominated by frontotemporal projections via the extreme and external capsules^44^. This ventral processing stream is commonly characterized as representing the “what” (object/goal recognition) of action perception^45^, including semantic tool knowledge^40,46^. In line with the broader concept of semantic memory^47^, such knowledge is considered semantic in the sense that it comprises abstracted or generalizable information (e.g., typical function, associative relationships) not tied to a specific instance, much as in word meaning and other forms of general world knowledge. In contrast to this relatively conserved ventral stream, frontoparietal projections via the middle and superior longitudinal fasciculi are better developed in chimpanzees and become quite pronounced in humans. Across these three taxa, there is thus a trend toward the elaboration of parietal inputs to IFG, in addition to robust pre-existing ventral stream connectivity. This dorsal stream of visual processing is thought to support the kinematic and spatial “how” of action perception/execution^45^, including tool actions^40,46^. It has thus been argued^22,40^ that the evolutionary elaboration of human frontoparietal connectivity enabled greater integration of such details with ventral stream action semantics^48^ in the service of complex skill learning and execution. The dorsal stream terminations in the parietal lobe represent regions that have enlarged in human evolution and have been linked to visuospatial capacity, technological integration, and language^49^.

Further dissecting this frontoparietal system, Hecht et al.^50^ found that the third branch of the superior longitudinal fasciculus (SLFIII) connecting inferior parietal and inferior frontal cortices shows increasingly robust and anterior extension into IFG from macaques to chimpanzees to humans, especially in the right hemisphere. This again parallels observed rIFG functional recruitment by stone-tool making, including posterior portions (*pars opercularis*/BA44) related to attention and control^31^ of action that respond to tool-making action sequence complexity^20^ as well as middle^51,52^ portions (*pars triangularis*/BA45) that exhibit functional connectivity with the default mode network and appear to be involved in social cognitive and emotional processes including mentalizing^31^. In fact, Hecht et al.^53^ found that individual chimpanzees with more human-like SLFIII connectivity to middle rIFG were more likely to succeed at mirror self-recognition, a classic test of self/other (i.e., “agency”) awareness that likely requires comparing internal motor commands and predicted outcomes (forward models) with observed sensory feedback^54^. Such predictive processing^55^ is thought to provide a unifying computational basis for motor control and social interaction^56^, including the development of imitation, perspective-taking, empathy, and mentalizing capacities^57,58^. This would potentially explain middle rIFG involvement in behaviors ranging from stone-tool making action execution^14,18^ and observation^15^ to false belief and reversal learning tasks^31^, as well as the functional relevance of enhanced dorsal stream inputs from parietal cortex.

Taken together, then, comparative neuroscience and neuroarchaeological evidence indicate that functional systems supporting stone tool making have undergone substantial change over human evolution, and that these changes may be relevant to a much wider range of distinctively human capacities, from social cognition to language. Specific evolutionary mechanisms underlying this pattern could include natural selection on genetic variation in technological aptitude^19,24^ as well as more extended^59^ interactions between plasticity, development, and non-genetic inheritance^22^ that are increasingly recognized in human evolutionary studies^60,61^. However, no prior research has addressed the neural traits underlying individual variation in stone- tool making aptitude, and only one relatively small (n = 6) study^62^ has investigated neuroplasticity during stone-tool making skill acquisition. These points are crucial to understanding the evolution of toolmaking abilities because inter-individual variation is the foundation on which natural selection acts, and intra-individual variation (i.e., acquired plasticity) is theorized to facilitate adaptive change^59^.

**Figure 1:**
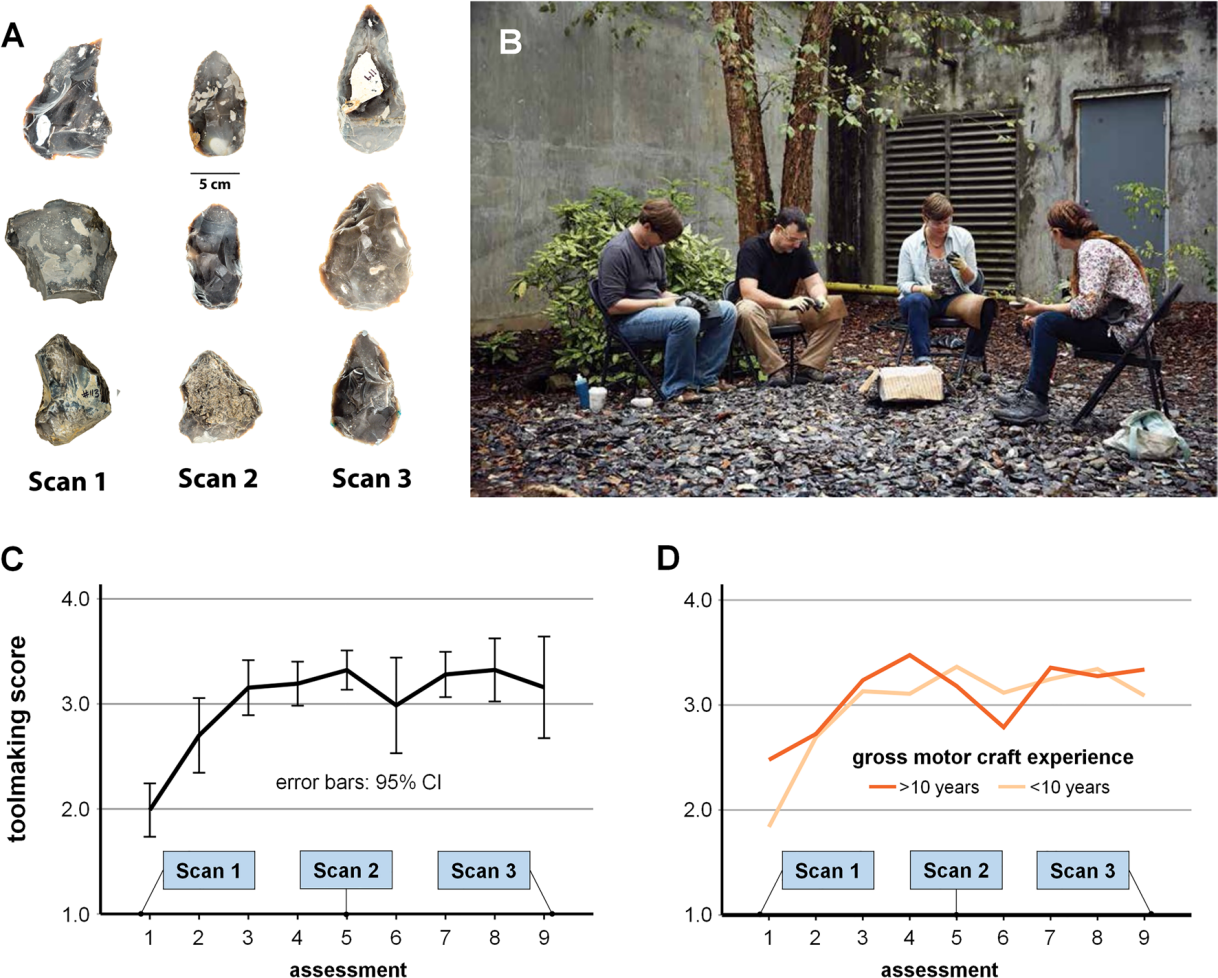
Participant artifacts and training. (**A**) Sample handaxes produced by participants for skill assessments at Scans 1, 2, and 3. (**B**) A practice session. Photo copyright Gregory Miller (gregorymillerpictures.com). (**C**) Learning curve in the whole trained group. (**D**) Learning curve separated by prior gross motor craft experience.

Accordingly, we initiated a multifaceted research project^10–12^ to investigate the acquisition of later Acheulean handaxe-making skills directly comparable to those documented at the ~ 500,000 year old archaeological site of Boxgrove in southern England^63,64^. Previous analyses of artifacts produced during this training program (Fig. 1) confirmed the presence of overall, group-level improvement as well as substantial individual variation in both initial performance and subsequent learning^10^. Results also confirmed the hypothesized^65^ importance of prior experience, finding that initial tool-making performance was correlated with self-reported years of experience in gross motor crafts like carpentry and sculpture^11^. Here we report results of a neuroimaging analysis using Diffusion Tensor Imaging (DTI) to measure white matter structure during this training program in 17 research participants, and in 16 control participants who received no training. Scans were collected before, at the mid-point, and at the termination of this archaeologically grounded, hands-on, Acheulean tool making program. This allowed us to examine neuroanatomical and experiential predictors of both the initial aptitude and subsequent neuroplasticity of individual participants, as well as group level training effects.

## Results

### A left frontotemporal pathway supports initial tool making aptitude

**Figure 2:**
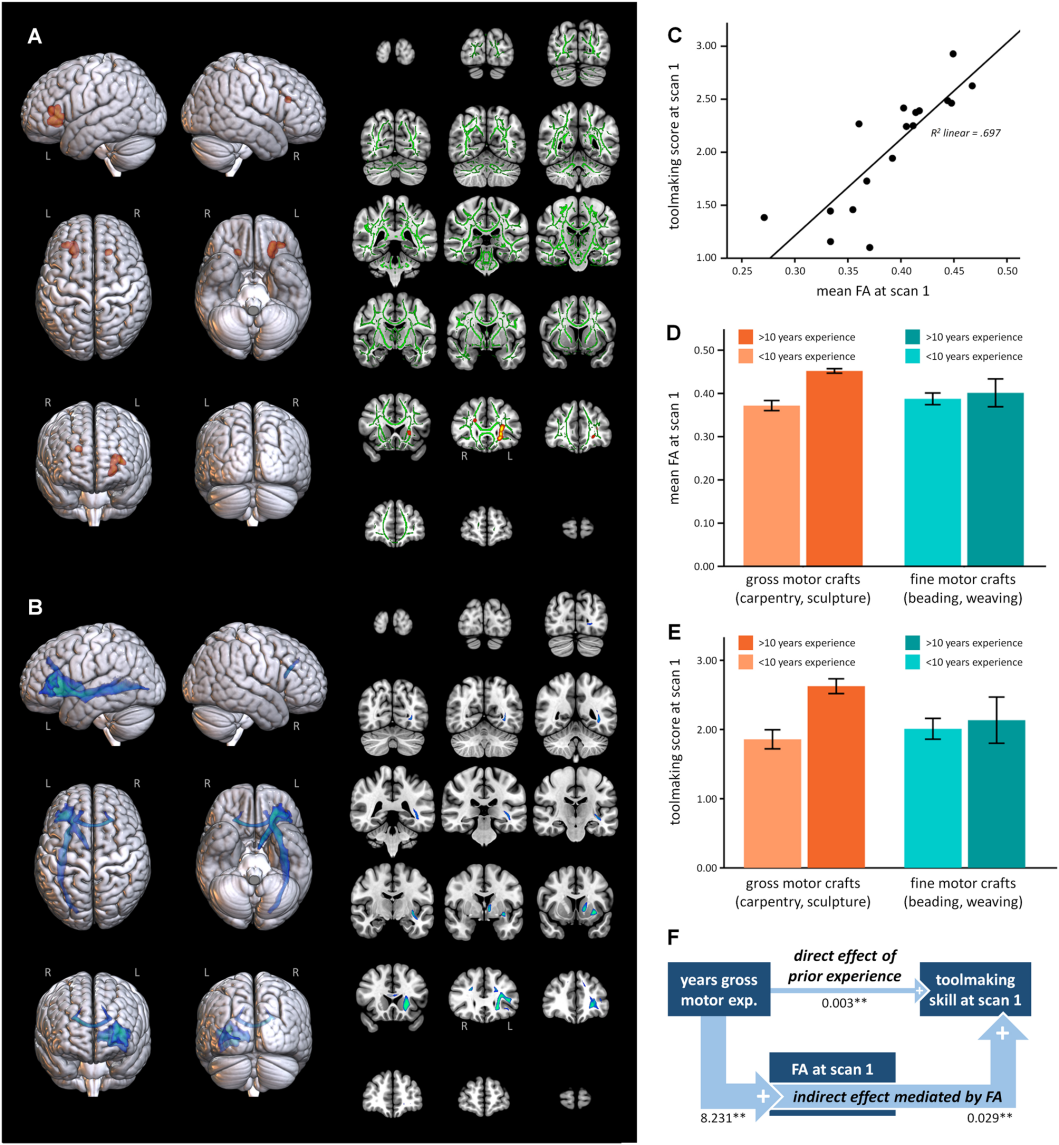
Baseline FA in ventrolateral prefrontal cortex is predictive of pre-training toolmaking performance. (**A**) Voxels showing a significant positive correlation between FA and toolmaking score at scan 1 are illustrated in orange. In 2D slices, the white matter skeleton is shown in green. (**B**) Probabilistic tractography from voxels showing a significant positive relationship between baseline FA and pre-training toolmaking scores. This is a composite image representing above-threshold connectivity in at least 67% of the participants. (**C**) Relationship between FA and toolmaking scores at scan 1 for the voxels indicated in (**A**). (**D**) Within these voxels, individuals with > 10 years of gross motor craft experience had significantly higher FA than subjects without this prior experience (t(15) = 2.3684, *p *= .002). This relationship did not hold for prior experience with fine motor crafts (t(15) = 0.465, *p *= .649). (**E**) Individuals with > 10 years of gross motor craft experience also had significantly higher toolmaking scores before training began (t(15) = 2.947; *p *= .010). Again, this relationship was not significant for fine motor craft experience (t(15) = 0.382; *p *= .708). (**F**) A causal mediation analysis showed that the effect of prior gross motor experience on pre-training toolmaking scores occurred almost entirely via the effect of prior experience on baseline FA.

Individuals who made better handaxes on their very first, pre-training attempt had higher fractional anisotropy (FA) in left ventrolateral prefrontal and right deep prefrontal white matter at scan 1 (Fig. 2A). Two large, near-contiguous clusters were located beneath middle and anterior IFG (BA45/47). Tractography revealed that these clusters connected with lateral temporal cortex via a ventral route with terminations (individual tract threshold of 0.001, group threshold of 67%) along the length of the Superior Temporal Sulcus (STS) (Fig. 2B). Anatomically, such fibers would likely be assigned to uncinate and extreme capsule fascicles within the ventral association tract system^66^.

The BA45/47 terminations identified in this result are anterior to the portion of IFG (*pars opercularis*, BA44) commonly highlighted in models of simple, everyday tool use^40,46^ and are thus expected to support more abstract and generalizable representations of actions, goals, and objects^67^ useful for learning novel tasks^68–70^. Consistent with this interpretation, we found that both ventrolateral prefrontal FA and initial tool making scores were influenced by years of prior experience with gross motor crafts like carpentry and sculpture (Fig. 2D-E). Participants with > 10 years of such experience had significantly higher FA and pre-training toolmaking scores than other participants (FA: t(15) = 3.679, *p *= 0.002; toolmaking: t(15) = 2.947; *p *= 0.010; both two-sided). These effects were not present for participants’ prior experience with fine motor crafts like beading, weaving, and felting (FA: t(15) = 0.465, *p *= 0.649; toolmaking: t(15) = 0.382; *p *= 0.708; both two-sided).

Because there was a significant correlation between participants’ age and years of prior experience with gross motor crafts (older participants had more years of experience; r = 0.707, *p *= 0.001, 2-tailed), we performed an additional regression analysis to ensure that age did not completely account for the observed relationship between tool making score and FA. The overall regression model was significant (F(2,14) = 23.114, *p *< 0.001). The beta coefficient for age was marginal (standardized beta = 0.275, *p *= 0.058), and the coefficient for tool making score was significant (standardized beta = 0.763, *p *< 0.001). The subjects with the 4 highest tool making scores were all over 40 years old, but this result also held if subjects over 40 years old were excluded from the analysis (F(2,9) = 10.213, *p *= 0.005; standardized beta for age = -0.075, *p *= 0.696; standardized beta for toolmaking score = 0.825, *p *= 0.002), indicating an effect of toolmaking skill on FA independent of age.

Importantly, the relationship between FA and pre-training tool making score was also evident in participants without prior craft experience (df = 10, F = 8.071, *p *= 0.019, Standardized beta = 0.688). This strongly suggests that the benefits of prior gross motor craft experience are mediated by its effects on relevant white matter anatomy. We tested this hypothesis using a causal mediation analysis in R version 4.0.3’s *mediation* package (Fig. 2F). The regression coefficient between years of gross motor skills and FA values and the regression coefficient between pre-training toolmaking scores and FA values were both significant. The indirect effect was (8.231)*(0.029) = 0.024. We tested the significance of this indirect effect using bootstrapping procedures. Unstandardized indirect effects were computed for each of 1,000 bootstrapped samples, and the 95% confidence interval was computed by determining the indirect effects at the 2.5th and 97.5th percentiles. The bootstrapped unstandardized indirect effect was 0.02, and the 95% confidence interval ranged from 0.01 to 0.06. Thus, the indirect effect was statistically significant (*p *< 0.001) and an order of magnitude greater than the direct effect of prior experience on pre-training tool-making performance, indicating that the effect of gross motor experience on model 1 test results was mediated via the measured white matter FA values.

Our results thus indicate that ventrolateral prefrontal FA predicts initial tool making success and is in turn influenced by prior experience. Our data do not allow us to determine if the association between FA and craft experience is a plastic effect of behavior or a pre-existing factor predisposing individuals to certain activities. However, the presence of a strong and significant association between years of experience and FA (logarithmic regression, r^2^ = 0.765, *p *= 0.023) across the six participants with > 10 years gross motor craft experience strongly suggests a plastic component.

The right hemisphere deep prefrontal white matter cluster connected to the left hemisphere ventrolateral prefrontal cluster via the corpus callosum (Fig. 2B). This may reflect the bilateral nature of cognitive control process in IFG^71^, the coordinated bimanual nature of stone tool-making actions^14^, and/or the integration of left hemisphere semantic representations with right hemisphere action regulation and body representation^31^. MNI coordinates and statistics for voxels with a significant positive correlation with tool making score at scan 1 are presented in Supplementary Table 2.

### A right frontoparietal pathway undergoes plastic change during tool making training

Whole-brain TBSS analyses indicated that at the group level, no regions of white matter showed significant shifts in FA during the course of training. This is in contrast with our results from an earlier study^62^. In the prior study, the sample consisted entirely of archaeology students from Exeter University aged 18–25 years. These participants were unlikely to have the same range of prior experience and initial aptitude captured by the current study. In addition, the training program included coordinated high-intensity training episodes (field trips) that likely aligned learning trajectories across individuals. In the current study, greater initial variability in prior experience, baseline tool-making skill, and white matter FA combined with less punctuated training may have produced more individually variable trajectories of neuroanatomical change during learning and made it difficult to detect a single consistent pattern of plasticity at the group level.

Accordingly, we sought to increase the sensitivity of our analysis by examining change in fiber orientations using FSL’s tbss_x tool, a method that aligns diffusion vectors across subjects^72^. Our rationale for this approach was that the anterior termination of the SLF in premotor and ventrolateral prefrontal cortex is a site of substantial crossing fibers. A number of fiber tracts meet in this region, including the SLF and arcuate fasciculus, interhemispheric connections via the corpus callosum, and the extreme capsule, uncinate fasciculus, and corticospinal tract. As a result, the primary fiber orientation in one subject may correspond to the secondary fiber orientation in another subject, and vice versa; plastic change in one fiber population but not another might therefore be difficult to detect, especially in the case of high variation across individuals, as the behavioral data on skill learning in the current study^10–12^ seemed to indicate.

**Figure 3:**
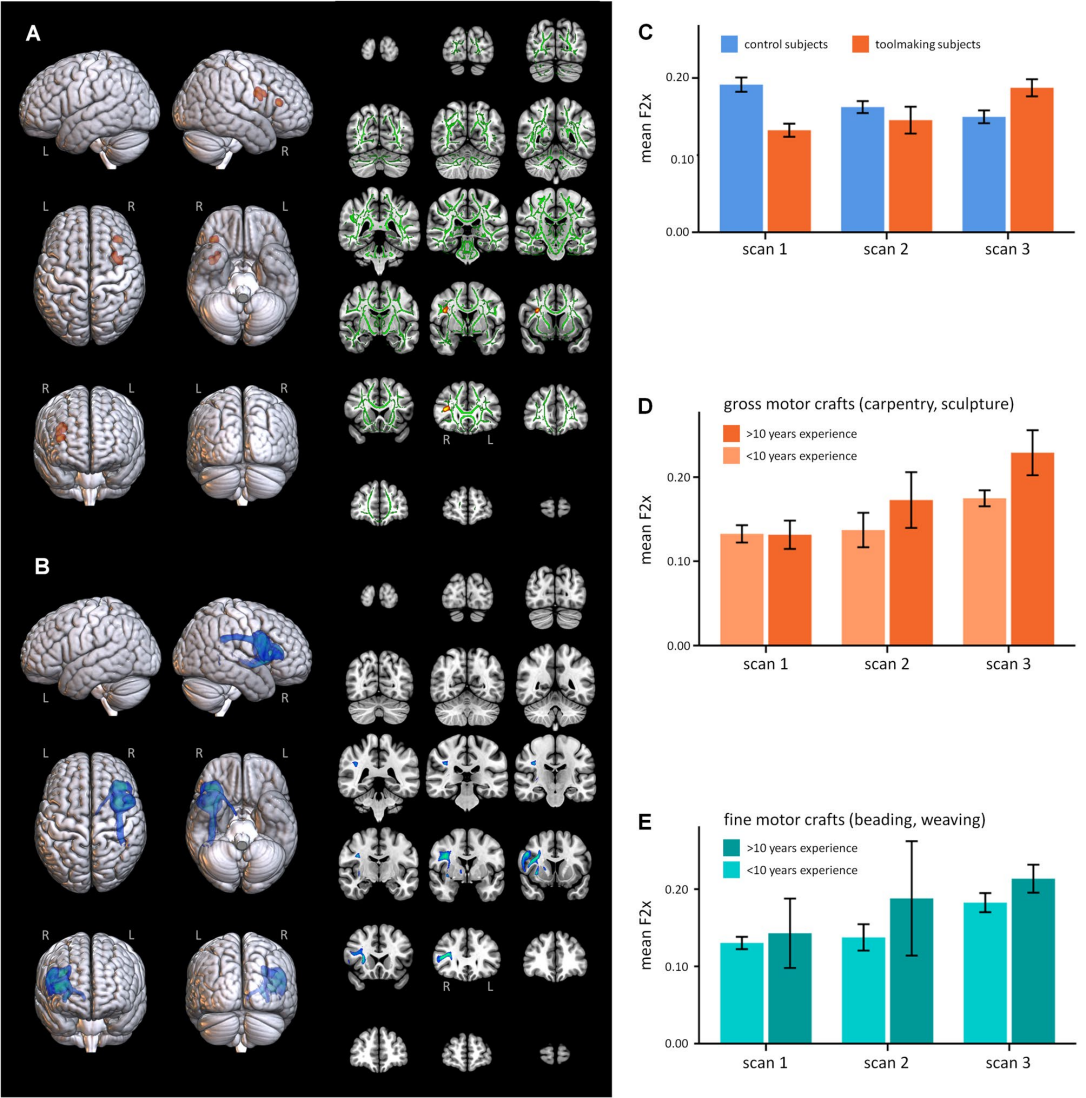
Neuroplastic change in the experimental group as compared to the control group. (**A**) Voxels illustrated in orange show a significantly more positive slope from scan 1 to scan 3 in the experimental group as compared to the control group in F2x (i.e., the estimated proportion of the diffusion signal that can be accounted for by the second fiber orientation after alignment of diffusion vectors across subjects). In 2D slices, the white matter skeleton is shown in green. (**B**) Probabilistic tractography from voxels showing neuroplastic change in (**A**). This is a composite image representing above-threshold connectivity in at least 67% of the participants. (**C**) Mean F2x value (i.e., the estimated proportion of the diffusion signal that can be accounted for by the second fiber orientation after alignment of diffusion vectors across subjects) at scans 1, 2, and 3 in the control and toolmaking groups. (**D**) F2x increased more rapidly in toolmaking participants with > 10 years of prior experience in gross motor crafts as compared to toolmaking participants without this prior experience, although no pairwise comparisons reached significance. (**E**) A similar pattern was visible in participants with > 10 years of prior experience with fine motor crafts.

Tbss_x in our sample aligned inferior frontal fibers into a primary orientation (F1x) corresponding to interhemispheric callosal connections and a secondary orientation (F2x) corresponding to SLF. We identified two clusters in white matter underlaying right ventral premotor (rPMv) and rIFG where F2x signal proportion increased in the tool making group vs. the control group (Fig. 3A; Supplementary 2). Voxels showing significant change were located within the anterior extension of the third branch of the superior longitudinal fasciculus (SLFIII) (Fig. 3B). In close agreement with prior structural^62^ and functional^14,15,18,20^ studies, tractography from voxels in these two clusters identified fibers linking the right inferior parietal lobe to cortical terminations throughout right PMv, posterior (BA44), and middle (BA45) IFG. This anatomical localization implicates a wide range of potential functions from premotor regulation of primary motor activity^73,74^ to IFG action execution, inhibition, spatial attention, mental reasoning, and social cognition^31^. F2x increase in the tool making group was significant from scan 1 to 3 (t(11) = 5.747, *p *< 0.001) and from scan 2 to 3 (t(11) = 2.683, *p *= 0.021), but not from scan 1 to 2 (t(15) = 0.758, *p *= 0.460; all two-sided), irrespective of an observed decrease in F2x over time in the control group (Fig. 3C).

Participants with > 10 years prior experience with gross motor crafts like pottery and carpentry showed stronger initial performance and plateaued earlier, with an apparent dip in performance around the midpoint of training that is suggestive of behavioral experimentation with new techniques (Fig. 1D). In keeping with this, these individuals also showed an earlier trend toward F2x increase from scan 1 to scan 2, higher F2x at scan 3 (t(11) = 2.450, *p *= 0.032) and a significantly greater (t(11) = 2.86, *p *= 0.016) overall increase by scan 3 (Fig. 3D). A similar pattern occurred in association with prior experience for fine motor crafts but did not reach significance (Fig. 3E). Thus, in addition to enhancing initial performance, *prior experience accelerated plasticity*. As with initial aptitude, this learning effect was associated with structural differences in the left hemisphere ventral frontotemporal action semantics pathway: scan 1 left ventrolateral prefrontal white matter FA significantly predicted the magnitude of right hemisphere F2x increase in individuals over the course of the study (F(12) = 7.418, *p *= 0.02, standardized beta = 0.635).

## Discussion

In this study, we measured white matter microstructure during the acquisition of Paleolithic stone tool making skill, an evolutionarily important behavior that is empirically well documented in the archaeological record. First, we sought to identify the structural basis of individual differences in tool making ability, as such phenotypic variation forms the raw material for evolution by natural selection^75^. Modern neurophenotypic variants associated with increased capacity for tool making learning are taken to indicate traits that would also have been adaptive in our species’ evolutionary history, even if evolutionary processes have shifted the species-typical range of variation. Second, we sought to identify plastic effects of tool-making experience in a larger sample of participants with diverse prior history with motor crafts, while maintaining consistent and well-controlled training experiences across the entire sample. Recently-enlarged human brain areas overlap with those that are particularly slow to develop^76^, suggesting that it is adaptive for these regions to remain plastic into adulthood. Given that stone-tool making is a learned skill that was practiced by human ancestors for > 2.5 million years, plasticity resulting from tool making skill acquisition is likely indicative of accumulated adaptive change. Finally, we sought to identify the role of prior experience with learned motor crafts in the acquisition of Paleolithic stone tool making. Understanding such potential interactions *between* culturally transmitted skills, and especially the neural mechanisms involved, is critical to unpicking the complex bio-cultural feedback dynamics thought to have driven human brain evolution^37,77–79^.

This study produced three major findings. The first of these is that individual variation in brain organization significantly predicted tool making aptitude, even before training began. We found that pre-training skill was positively associated with white matter FA beneath the middle and anterior portions^51,52^ of left IFG (cf. BA 45/47) (Fig. 1A, 1C). These voxels were located within a ventral tract connecting inferior frontal with temporal cortex, a pathway commonly associated with semantic processing for both language^80,81^ and tool use^46,66^ (Fig. 1B). The frontal and temporal targets of this tract are functionally complex regions^82,83^ and the ventral pathway identified here overlaps with frontotemporal circuits believed to support semantic processing for both language^80^ and action understanding^67^, including tool use specifically^46^. We thus propose the hypothesis that the association between initial tool quality and left ventrolateral prefrontal FA reflects the influence of structural variation in this pathway on individual differences in action semantic processing, including the representation of abstract functional and associative relationships between tools, actions, and goals^46,67^. This might include actual linguistic encoding^18,68,69,84^ and/or similar cognitive operations on non-linguistic representations in a spatially overlapping, parallel pathway. Such semantic representation enhances generalizability and facilitates both the motor learning of new tools^69^ and the analogical reasoning that allows application of familiar concepts to novel tasks^68,70^. Structural variation in this pathway may thus hypothetically provide an anatomical basis for individual aptitude in acquiring novel technical/craft skills like stone tool making. This would identify one specific mechanism and associated neuroanatomical target of selection (proximate basis and adaptive function, sensu Tinbergen) for the evolution of a more general human technological learning capacity^35,85^.

**Figure 4:**
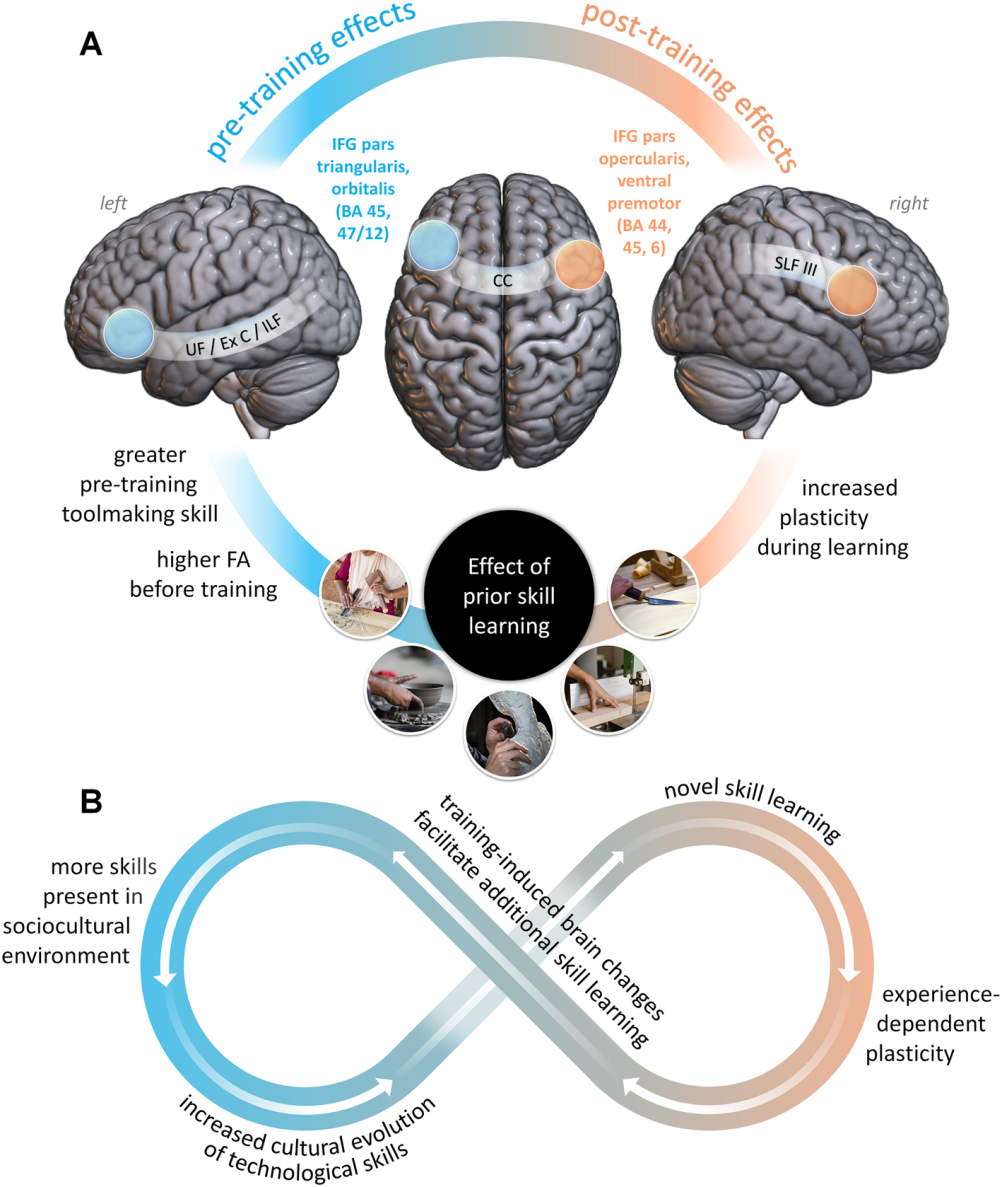
(**A**) Summary diagram of the results of this study. (**B**) Schematic of hypothetical bio-cultural feedback loop by which “skill begets skill” – acquisition of new technological skills exerts plastic effects on brain anatomy, which enhance technological learning abilities, thereby facilitating further cultural evolution of those skills and promoting further skill learning.

FA in these voxels was itself associated with prior experience with other gross motor crafts such as pottery and carpentry (Fig. 1D). Furthermore, this prior experience significantly increases pre-training toolmaking ability via effects on FA in the measured voxels (Fig. 1E-F). This suggests the possibility that white matter structure in a ventral frontotemporal pathway may provide an anatomical basis for “learning to learn”^86,87^ certain kinds of tasks through the generalization of relatively abstract and context-independent semantic representations. Confirming this possibility with additional research could have direct practical relevance for modern human skill acquisition, but also has intrinsic importance for our understanding of our own species. Putatively, this could provide an additional mechanism for bio-cultural feedback in which the cultural evolution and behavioral adoption of new Paleolithic technologies exerted plastic effects on brain anatomy that enhanced technological learning capacities and thus facilitated further cultural evolution (Fig. 4B).

It is possible that this effect of prior experience is at least to some extent a result of intrinsic individual differences. Individuals who have a natural affinity for a certain type of visuomotor processing may be more likely to have devoted substantial time to gross motor crafts in the past and may also be more likely to volunteer for, and succeed at, Paleolithic stone toolmaking training. Prior single-timepoint neuroimaging studies examining white matter correlates of individual behavioral differences have found measurable effects within frontoparietal tracts for traits relevant to the current study such as bimanual coordination^88^, handedness^89^, and visual attention^90^. However, the fact that FA strongly co-varied with actual years of experience even among dedicated (> 10 years experience) craftspeople strongly suggests a contribution of experience-dependent plasticity.

This would be consistent with a large body of research focused on other learned skills which has established that skill learning produces structural plasticity in the adult human brain (reviewed in^91,92^). Perhaps most prominently, over 2 decades ago, enlargement of the posterior hippocampus was reported in London taxi drivers, who experience rigorous demands on spatial memory, in comparison with controls^93^. These results might conceivably be attributed to intrinsic individual differences; perhaps people with better navigation abilities are simply more likely to become taxi drivers. However, later studies ruled out this possibility^94^ and established that plastic change really does occur during successful training for the taxi driver exam^95^, in which drivers must spontaneously recall efficient A-to-B routes for arbitrary pairs of locations across the entire complexity of London’s 25,000 streets. Furthermore, this line of research established that prior skill learning has an impact on future skill learning: taxi drivers show a deficit at acquiring new visuo-spatial information^96,97^. Experience-dependent plasticity has also been reported for a number of other learned tasks. This includes plasticity inferred from cross-sectional studies on groups varying in prior skill training/practice (e.g.,^98–101^), as well as directly observed plasticity in longitudinal examinations for skills like learning a second language^102^, learning to juggle^103^, and training on a seesaw-like balance task^104^. Interestingly, pre-training brain measures also predict skill at this balance task^105^. Together, this accumulated evidence suggests that in the current study, participants with substantial prior gross motor craft experience likely underwent experience-dependent restructuring of brain networks prior to the onset of tool making training. Thus, the neuroanatomical variation driving individual differences in tool making aptitude may itself be a product of prior experience.

The second major finding of this study concerned training-related plasticity during stone-tool making skill acquisition. We observed changes to white matter in SLFIII under right PMv and IFG from Scan 1 (pre-training) to Scan 3 (post-training) (Fig. 2A-B). SLFIII connects inferior frontal and parietal cortex, and the localization of the current effect closely matches prior structural^62^ and functional^14,15,18,20^ studies implicating right inferior frontoparietal cortex in stone-tool making. The observation that this structural change was significant between scans 1 and 3, and between scans 2 and 3, but not between scans 1 and 2 indicates that structural remodeling in this pathway was predominantly associated with later stages of skill learning. Pargeter et al.^10^ showed that learning in our tool making group followed a roughly asymptotic curve, with rapid initial gains leveling off at a performance plateau prior to the midpoint scan (Fig. 1C). This plateau remained well short of the benchmark performance of modern experts and the quality of actual Palelolithic handaxes from the Boxgrove collection. Such plateaus are common during skill learning and are thought to occur when incremental learning strategies converge on a sub-optimal strategy (e.g., “hunt-and-peck” visually guided typing)^106^. Plateaus represent periods of active skill consolidation and experimentation allowing a subsequent transition to more optimal strategies (e.g., touch typing), although this transition may involve a temporary dip in performance as a new skill set is acquired^106^. In handaxe making, early-stage learners typically focus on simply approximating the classic teardrop shape of the handaxe before transitioning to the more demanding strategies required to simultaneously thin the cross-section and achieve expert performance^63^. Pargeter et al.^10^ thus concluded that the observed performance plateau reflected an active learning period of perceptual-motor consolidation and behavioral experimentation even though no group-level performance increase was evident. We now find that this plateau is associated with plastic enhancement to right frontoparietal connectivity via SLFIII, thus identifying a neuroanatomical signature for this learning stage. This is consistent with prior evidence of right SLFIII remodeling induced by stone-tool making practice^62^ and the functional interpretation of this tract as contributing to refined action control and bodily awareness. An important target for future research will be to address the separate and combined roles of sensory, motor, and cognitive learning during toolmaking skill acquisition, as these processes undoubtedly interact and cannot be examined individually in the current study.

The third major finding of this study was that past skill experience significantly impacts neural mechanisms of new skill acquisition. Among participants who received tool making training, prior experience with gross motor crafts like pottery and carpentry was significantly linked, not only with initial aptitude, but also to the magnitude of training induced plastic change in right frontal white matter (Fig. 2C-E). In participants with > 10 years of such experience, the amount of plastic change within these voxels steadily increased from scan 1 to scan 3 and significantly outpaced participants without this experience by the end of the study. These voxels were located in the superior longitudinal fasciculus, beneath ventral premotor cortex and the pars opercularis (BA44) and triangularis (BA45) of the inferior frontal gyrus. Interestingly, the gray matter of the inferior frontal gyrus is linked to both crystallized and fluid intelligence^107^, while the SLF is linked to fluid intelligence^108^. This further supports the interpretation of this left frontotemporal tract as providing an anatomical basis for abstracting and generalizing experience in order to “learn to learn” similar tasks, leading to accelerated right frontoparietal plasticity involved in the subsequent acquisition of more refined context-specific action regulation and embodied skill. These possibilities represent an important target for future research. A similar pattern occurred in association with prior experience for fine motor crafts but did not reach significance, suggesting that the effect of past skill learning is more pronounced for past skills that are more similar to the new skill. Together with the link between prior experience and pre-training toolmaking skill, these findings indicate that prior experience has a measurable impact both on how individuals initially approach the challenge to learn a new technical skill, and on the trajectory of plasticity their brains undergo as they learn it (Fig. 4A), in effect documenting a process of “learning to learn”^86,87^ technical skills. Such experience-based facilitation is thought to reflect the abstraction of generalizable regularities of task structure^109,110^, which is consistent with the localization of our aptitude effect to a frontotemporal action-sematic pathway.

Speculatively, this could have an important implication for human evolution. If individuals’ efficacy or efficiency at learning new skills is influenced by the prior skills they have learned, and this effect is mediated by plastic neuroanatomical accommodation, this creates a situation in which plasticity-led neural adaptations^59,111^ for one behavior could be readily co-opted (“exapted”^112^) to facilitate the discovery and social transmission of additional, related skills. Such a dynamic would provide one specific mechanism for the autocatalytic feedback between brain and cultural evolution suggested by formal models^37^ and phylogenetic comparative analyses^113^. In other words, at times and places where more and more object-manipulatory, tool use, and tool making skills were socially learned and culturally transmitted within and between groups, the addition of further learned skills may have become easier and easier. This is a speculative idea, but it has an easily testable implication: it suggests that “skill begets skill”, i.e., that acquisition of one technical skill should facilitate acquisition of other skills. Such facilitation has been documented with simpler perceptual and motor skills (e.g., joystick aiming^86^, shape categorization^110^) and used to explain the more general cognitive benefits of action video game playing^87^. In nonhuman animals, the cross-modal transfer of learned responses based on sensory associations have long been a topic of study (e.g.,^114–117^), and in domestic dogs, which are often engaged in skill learning to support human goals, individual animals who are highly trained perform better on a novel problem solving task^118^. In modern humans, second language learning facilitates third language learning (reviewed in^119^), and there is also evidence of skill transfer between music and speech (reviewed in^120^), and even between simple tool use and language^121^. Our results suggest that a similar process of facilitation could apply to technical skills, specifically including tool making abilities pivotal to the evolution of our species. If true, this idea could represent a behaviorally driven, feed-forward neuroanatomical mechanism contributing to increases in culturally transmitted technological skills and brain size over time (Fig. 4B).

## Conclusion

Taken together, these results identify likely neuroanatomical targets of selection on tool-making ability, document plastic response properties of these targets that would enable extended evolutionary-developmental processes, and link neural mechanisms of toolmaking skill acquisition to more general, fundamental neurocomputational processes supporting behaviors ranging from language to mentalizing. Researchers have long posited that human cognitive and brain evolution relied on exaptive, co-evolutionary, behavior-led feedback loops linking learning and adaptive change^37,78,79,113,122^. This study identifies specific brain-behavior mechanisms that may underlie these longstanding ideas.

## Methods

### Participants

Human research procedures were reviewed and approved by the Emory University Institutional Review Board (study 00067237). All participants provided written informed consent and all research was performed in accordance with relevant guidelines and regulations. Subjects were recruited from Emory University (students and staff) and the surrounding community following an intensive advertising campaign. Participants in the experimental group received toolmaking training as described below. Participants in the control group received no such training but did receive scans at the same time intervals. Participant details are shown in Table 1. 17 toolmaking and 16 control participants completed scans 1 and 2. Four participants from the toolmaking group (6, 8, 13, and 15) and 3 participants from the control group (29, 30, and 38) dropped out of the study prior to scan 3.

**Table 1:** Participant details.

Participant	Group	Age (years)	Gender	Prior experience with gross motor crafts (years)	Prior experience with fine motor crafts (years)
01	Toolmaking	34	Male	0	0
02	Toolmaking	22	Female	0	0
03	Toolmaking	27	Female	0	3
05	Toolmaking	45	Female	0	1
06	Toolmaking	43	Male	20	20
07	Toolmaking	36	Female	0	10
08	Toolmaking	23	Female	0	2
09	Toolmaking	29	Female	0	0
10	Toolmaking	23	Female	0	5
11	Toolmaking	42	Male	15	10
13	Toolmaking	19	Female	3	8
14	Toolmaking	49	Male	35	5
15	Toolmaking	30	Female	0	15
16	Toolmaking	43	Male	30	3
17	Toolmaking	25	Female	0	5
19	Toolmaking	24	Female	0	0
21	Toolmaking	24	Male	7	0
22	Control	23	Female		
23	Control	21	Female		
24	Control	43	Female	3	15
25	Control	18	Female	2	0
26	Control	23	Female		
27	Control	21	Female	0	5
28	Control	20	Female		
29	Control	27	Female	0	1
30	Control	27	Female	3	6
31	Control	31	Female	21	21
32	Control	31	Male	0	0
33	Control	21	Female		
34	Control	20	Male	0	0
35	Control	37	Female	0	7
37	Control	20	Female		
38	Control	22	Female		

### Tool-making training and testing

Training was provided by Nada Kreisheh, an experienced knapping instructor^123^, with 10 years of knapping practice and knowledge of Late Acheulean technology. The experiment aimed to test the participant's ability to learn the process of Late Acheulean style handaxe production including how to select appropriate toolkits, initiate flaking on a nodule, maintain the correct flaking gestures and angles, visualize outcomes, deal with raw material imperfections, and correct mistakes. Full participation in the study amounted to ~ 90 h of which ~ 80 h involved training in handaxe production. Participants were given formal learning assessments at 10-h increments over the training program. Each participant's resulting handaxe was scored on a 5-point scale using a multivariate model designed to grade standard technical criteria^10^.

Prior experience was assessed by self-report on an open response questionnaire asking participants to “list any craft skills (e.g. carpentry, knitting, basketry, etc.) you have practical experience of” along with the “number of years practiced.” Participants listed eighteen different craft skills ranging from beading to welding. These responses were aggregated for analysis as “gross” or “fine” motor crafts based on their involvement of large limb and object movements vs. smaller-scale manual manipulation.

### Image acquisition

Scanning occurred at Emory University using a Siemens Magnetom PrismaFIT 3 T. Image sets used in the present analysis included T1-weighted structural MRI and a 91-direction diffusion-weighted sequence with 7 B0-weighted images collected in the anterior–posterior phase encoding direction. An additional 5 B0 images were collected with reverse phase encoding to allow for EPI unwarping. Voxel size was 1.00 mm^3^ isotropic for T1 images and 1.25 mm^3^ isotropic for diffusion images.

### Image pre-processing

Two free, open-source software packages, the FMRIB Software Library (FSL)^124–126^ and Advanced Normalization Tools (ANTs)^127,128^ were used for image processing. T1-weighted images were skull-stripped using BET^129^, bias-corrected using FAST^130^, and then nonlinearly aligned to the MNI template using ANTs. For diffusion-weighted images, EPI distortion was accomplished with topup^131^, eddy current correction was accomplished using eddy^132^. Diffusion tensors were fit using DTIFIT, and a probabilistic distribution of fiber orientations was calculated using bedpostx, both part of FSL’s FDT toolkit^133,134^. Fractional anisotropy (FA) images were nonlinearly aligned to the FMRIB 1 mm FA template using ANTs.

### Image analysis

We used an in-house version of FSL’s TBSS processing pipeline^135^ which was amended to rely on ANTs-based registrations. After all subjects’ FA images were nonlinearly aligned to the FMRIB FA template, a mean FA image for the entire dataset was computed. FA values were projected onto white matter cores using FSL’s *tbss_skeleton* command. Individual subjects’ FA data were projected onto this mean FA skeleton and thresholded at FA > 0.125. Finally, these images were subjected to general linear modeling and Monte Carlo permutation testing using *randomise*, with the significance threshold set to *p *< 0.05 after multiple comparisons correction via threshold-free cluster enhancement^136^. For tractography, *tbss_deproject* was used to map significant MNI-space voxels back to subjects’ native diffusion space. We used the following parameters for *probtrackx2*: loopcheck on; curvature threshold 0.2; 2000 steps per sample; steplength 0.5; fiber threshold 0.1; 5000 samples per seed voxel. Path distributions were thresholded at 0.1% of the waytotal, binarized, and warped back to MNI space using ANTs, and summed in order to create template-space composite images. In these composite images, voxel value represents the number of subjects with above-threshold connectivity at that point. Composite images were thresholded at the group level to only show voxels where at least 67% of subjects had above-threshold connectivity.

## Supplementary Information

Supplementary Information.

## Data Availability

All stone tool data and associated R code used to generate the skill metric analyzed during the current study are available in the Open Science Framework repository https://osf.io/h5c8t/. All other behavioral, questionnaire, and neuroimaging data generated or analyzed during this study are included in this published article and its supplementary information file.
